# Protective Effects of *Cydonia oblonga* Mill. Fruit on Carbon Tetrachloride-induced Hepatotoxicity Mediated through Mitochondria and Restoration of Cellular Energy Content

**DOI:** 10.22037/ijpr.2020.112534.13812

**Published:** 2020

**Authors:** Maryam Noubarani, Shaghayegh Abaei Khayat, Romina Mafinezhad, Mohammad Reza Eskandari, Mohammad Kamalinejad, Sina Andalib, Shohreh Mohebbi

**Affiliations:** a *Department of Pharmacology and Toxicology, School of Pharmacy, Zanjan University of Medical Sciences, Zanjan,* *Iran. *; b *School of Pharmacy, Shahid Beheshti University of Medical Sciences, Tehran,* *Iran. *; c *Department of Medicinal Chemistry, School of Pharmacy, Zanjan University of Medical Sciences, Zanjan, Iran.*

**Keywords:** ATP, HPLC, Hepatic regeneration, Hepatoprotection, Mitochondria, Quince

## Abstract

Quince (*Cydonia oblonga* Mill.) is one of the medicinal plant with a broad range of pharmacological activities such as hepatoprotective effect. The present study was conducted to evaluate the effect of aqueous extract of *Cydonia oblonga* Mill. fruit (ACOF) against carbon tetrachloride (CCl_4_)-induced liver damage in rats. Hepatotoxicity was induced by CCl_4_ and all tested group animals were treated with the plant extract at a dose of 75, 150, and 300 mg/kg orally for 5 days. Blood was collected for the assessment of serum marker enzymes (alanine transaminase (ALT), aspartate transaminase (AST), alkaline phosphatase (ALP), and lactate dehydrogenase (LDH)). Adenosine triphosphate (ATP) of liver mitochondria was also measured using a validated high performance liquid chromatography (HPLC) method. The antioxidant capacity of the extract resulted in the reduction of MDA and the restoration of GSH in the liver (*P *< 0.05). Free radical scavenging activity of the extract was evaluated by DPPH method and the IC_50_ value was found to be 568 μg/mL. Our results indicated that bioenergetic depletion occurred in the intoxicated rats as a consequence of mitochondrial dysfunction and ATP production collapse. ACOF markedly restored ATP contents that is a key step in liver regeneration. It can be concluded that the role of ACOF to improve liver function on CCl_4_-hepatoxicity could be attributed, at least partially, to its action at mitochondira by preventing the loss of ATP content.

## Introduction

 Liver injury due to chemicals or infectious agents may lead to progressive liver fibrosis and ultimately cirrhosis and liver failure. However, no efficient drug that delays progression of liver damage, improves liver functions otherwise facilitating liver regeneration is not available. Nowadays, efforts are being performed worldwide to get scientific supports for traditionally used hepatoprotective herbals (1-5). 

*Cydonia oblonga *Mill (commonly known as quince) is a medicinal plant from Rosaceae family with broad range of pharmacological activities attributed to various parts of quince (6-13). In the previous studies hepatoprotective potential of the quince leaves has been reported (14, 15). Moreover, we have previously reported the hepatoprotective activity of the aqueous extract of *Cydonia oblonga* Mill. fruit (ACOF) in diabetic rats as well as its chemopreventive effect against hepatocellular carcinoma in rats (16, 17). 

Based on our previous results and quince usage as effective agent in the management of liver diseases in Iranian traditional medicine, the present study was undertaken to investigate the effect of ACOF on CCl_4_-induced oxidative damage in rat liver (18). 

The mitochondrial damage is the critical step in liver injury as mitochondria have central role in the cellular energy production. The energy state of the cell is a vital requirement for initiating liver recovery (19). Therefore, quantifying hepatic mitochondrial ATP content could be also useful in the selection of herbal extracts or any treatment for further investigations to improve liver function as hepatocellular regeneration that affects the final outcome of liver injury.

The precise determination of ATP is essential in the study of energy metabolism. Bioluminescence methods are the most sensitive and widely used in the determination of ATP in different matrices. However, this technique is expensive because of the use of luciferin–luciferase; therefore, it is inappropriate for routine analyses. HPLC method is less sensitive but more selective, chiefly, due to grouping of separation and determination stages. Besides, the short time of analysis and the precise assessment of ATP make this method worthwhile for routine use. However, in the most hepatotoxicity studies, ATP changes have been ignored due to expensive or time waste process. Therefore, additionally the effect of ACOF against mitochondrial adenosine triphosphate (ATP) depletion in hepatotoxicity induced by CCl_4_ was assessed.

##  Experimental

Carbon tetrachloride (CCl_4_) and silymarin were obtained from Sigma-Aldrich Co. (Taufkrichen, Germany). Adenosine 5-triphosphate disodium salt (ATP) was purchased from Sigma Chemical Co., St. Louis, USA. Perchloric acid 70% solution in water (PCA) and Tetrabutylammonium hydrogen sulphate (TBAHS) were purchased from Sigma–Aldrich, Steinheim, Germany. Potassium dihydrogen phosphate (KH_2_PO_4_), potassium carbonate (K_2_CO_3_), and di-potassium hydrogen phosphate trihydrate (K_2_HPO_4_·3H_2_O) were obtained from Merck, Darmstadt, Germany. HPLC-grade methanol was purchased from Scharlau (Spain). All other utilized chemicals were of analytical grade. 

Quantification of ATP was performed using a Knauer HPLC system consisting of a binary gradient pump, an adjustable wavelength detector (set at 254 nm) and a solvent degasser system. Separation was attained with a 3 µm particle size Supelcosil LC-18-T column (150 mm × 4.6 mm i.d.); Sigma-Aldrich company (USA) was protected by a 5 µm Supelguard C18 guard column (20 mm × 2.1 mm i.d.; Sigma-Aldrich).

*Plant resources and preparation of the extract*

*Cydonia oblonga* Mill. fruits were collected from Shahriar, Alborz province, Iran. The collected fruits were scientifically approved by the Department of Botany, Shahid Beheshti University of Medical Sciences and a voucher specimen was deposited in Shahid Beheshti University of Medical Sciences Herbarium (8054). The cleaned fresh fruits with their peels were dried in the shade at room temperature. Extraction of dried fruits was carried out by maceration with water for 30 min. Finally, the extract was filtered and was kept at –20 °C until use. The extract was dissolved in distilled water to receive desired concentrations just before use. The moisture level of the extract was determined by weight loss after placing 2 g of the final extract in an oven at 60–65 °C for 72 h. The final extract contained 24% water. 

*Standardization of Extract*

The total polyphenol content of the extract was measured using gallic acid as the standard based on the Folin–Ciocalteu method as previously has been described by Blainski (20). The total polyphenol content is expressed in mg of gallic acid equivalents (GAE) per g of the extract. The flavonoids content was measured using a colorimetric assay developed previously (21). The total flavonoid content is expressed in mg of quercetin equivalents (QuE) per g of the extract. 

Total polyphenolic content of ACOF calculated from the calibration curve (R^2^ *=* 0.991), was 43.42 ± 1.18 mg of GAE/g of the extract and total flavonoid content of ACOF calculated from the calibration curve (R^2^ = 0.996), was 13.63 ± 1.86 mg of QuE/g of the extract. The given values are mean ± SD of three different determinations.

*In-vitro antioxidant activity*

The DPPH free radical scavenging activity of ACOF was performed using BHT as a positive control (22). Briefly, the mixtures of methanolic solution of DPPH (40 µgmL^-1^) and different concentrations of ACOF (50-1000 µgmL^-1^) were prepared and the temperature was kept at 30 °C. The radical scavenging effect was constantly monitored by observing the alteration of absorbance at 517 nm for 30 min, against a blank (ACOF dissolved in methanol).

The activity percentage was calculated according to the following equation:

Scavenging% = [Abs. (control) – Abs. (mixture)]/Abs. (control) × 100 

Abs. (control) = Absorbance of DPPH solution (40 µgmL^-1^) at 517 nm

Abs. (mixture) = Absorbance of DPPH solution (40 µgmL^-1^) at 517 nm with 50-1000 µgmL^-1 ^of ACOF.

BHT (butylhydroxyotoluene) was used as positive control for comparison. IC_50 _value is the concentration of ACOF which is required to be reduced by 50% the primary amount of DPPH. It was calculated by a graph plotting percentage inhibition against concentration.

*Animal treatment and hepatotoxicity induced by CCl*_4_

Thirty-six male Sprague-Dawley rats weighing 180-200 g were housed in clean polypropylene cages with 12 h light/dark schedule, an environmental temperature of 21–23 °C, and a relative humidity of 50–60%. The animals were fed with normal pellet chow diet and given tap water ad libitum. The study was conducted according to principles of laboratory animal care (NIH publication No. 85-23, revised 1985) and the study protocol was approved by the Committee of Animal Experimentation of Zanjan University of Medical Sciences, Zanjan, Iran (ZUMS.REC.1394.208). The rats in each group (1-4, n = 6) received the treatment as described below:

Group 1 (normal control): single dose of water (1 mL/kg, p.o.) daily for 5 days + liquid paraffin (1 mL/kg, i.p.) on day 2 and 3.

Group 2 (CCl_4_-intoxicated): CCl_4_ (1 mL/kg, p.o.) once daily for 5 days + CCl_4_: liquid paraffin (1:1, 1 mL/kg, i.p.) on day 2 and 3.

Group 3 (standard): silymarin (50 mg/kg, p.o.) once daily for 5 days + CCl_4_ (as CCl_4_-intoxicated group) after 30 min of silymarin administration.

Groups 4–6 (extract treated): extract in doses of 75, 150, and 300 mg/kg (p.o.) once daily for 5 days + CCl_4_ (as CCl_4_-intoxicated group) after 30 min of the extract administration. The selection of the extract doses was based on our previous study (23).

The rats were sacrificed 24 h after the last treatment and the liver was dissected out and liver mitochondria were isolated by differential centrifugation (24). Mitochondria were prepared freshly and used within 4 h of the experiment, and all the steps were strictly operated in ice to guarantee the isolation of high-quality mitochondrial preparation. Five-hundred microliter mitochondria sample was processed as mentioned in the extraction procedure section.

*In-vivo antioxidant activity*

The level of malondialdehyde (MDA) as a reliable marker of lipid peroxidation was evaluated by TBA test (Smith *et al.*, 1982). Briefly, the MDA (the final product of lipid peroxidation) in the sample reacts with thiobarbituric acid (TBA) to produce a MDA:TBA adduct. The generated adduct is simply quantified with an ELISA reader instrument (Infinite M200, TECAN). MDA levels were presented as μg/mg protein.

Hepatic GSH contents were estimated in liver homogenate by a spectrophotometric method using DTNB as the indicator of GSH and expressed as µg/mg protein. The intensity of the yellow color produced in the samples was recorded at 412 nm with a UV spectrophotometer (Infinite M200, TECAN) (25). 

*Serum biochemical assays*

 The serum levels of alanine transaminase (ALT), aspartate transaminase (AST), alkaline phosphatase (ALP), lactate dehydrogenase (LDH), and total bilirubin were determined by commercially available enzyme kits (Pars Azmoon, Tehran, Iran) and using an automatic analyzer (Architect c8000 Clinical Chemistry System, USA) (26).

*Sample preparation for HPLC analysis*

 In the first step of preparation, 500 microliter of homogenized isolated mitochondria was mixed with 500 μL of Trichloroacetic acid 20% and vortexed for 5 min. Afterward, it was centrifuged at 4 °C at 12000 rpm for 10 min. in the next step, 650 μL of the supernatant was mixed with 20 μL of KOH (6 molar) and 20 μL of K_2_CO_3 _(2 molar) and vortexed. Then, the solution was centrifuged at 4 °C at 12000 rpm for 10 min to separate the precipitate from the solution entirely and 50 µL of the supernatant was injected directly into the HPLC system.

*Chromatographic conditions*

Two buffers were used: buffer A (65 mM potassium phosphate buffer contained: 39 mM K_2_HPO_4_ and 26 mM KH_2_PO_4_, adjusted to pH 6 with orthophosphoric acid and 4 mM TBAHS) and buffer B (65 mM potassium phosphate buffer contained: 39 mM K_2_HPO_4_ and 26 mM KH_2_PO_4_, adjusted to pH 6 with orthophosphoric acid and 25% methanol). The flow rate was 1 mL/min, and the gradient profile used was as follows: 1 min 100% buffer A, 3 min to 30% buffer B, 7.5 min to 80% buffer B, and 10 min to 100% buffer B. The run was held at 100% buffer B for an extra 3 min and the gradient was switched back by 100% pumping buffer A. A 10-min reequilibration between runs was appropriate to restore initial conditions. 

*Histopathology*

The liver tissue was dissected out and fixed in 10% formalin, dehydrated in gradual ethanol (50–100%), cleared in xylene, and embedded in paraffin. The sections were prepared and then stained with hematoxylin and eosin (H&E) dye for photomicroscopic observation, including cell necrosis, fatty change, hyaline regeneration, and ballooning degeneration.

*Statistical analysis*

The difference among means has been analyzed by one-way ANOVA followed by Tukey’s HSD as the *post-hoc* test. A value of *P *< 0.05 was considered as statistically significant. All statistical analyses were done using commercially available software (Microsoft Office Excel 2003, v. 5.1; Microsoft, Redmond, Washington).

## Results and Discussion

*In-vitro antioxidant activity of ACOF*

 A significant decrease in the concentration of DPPH radical due to scavenging ability of the different concentrations of the extract (R^2 ^= 0.906) was exhibited. The IC_50_ of the extract was found to be 568 ± 2.1 μg/mL.

*In-vivo antioxidant activity of ACOF*

**Figure 1:**
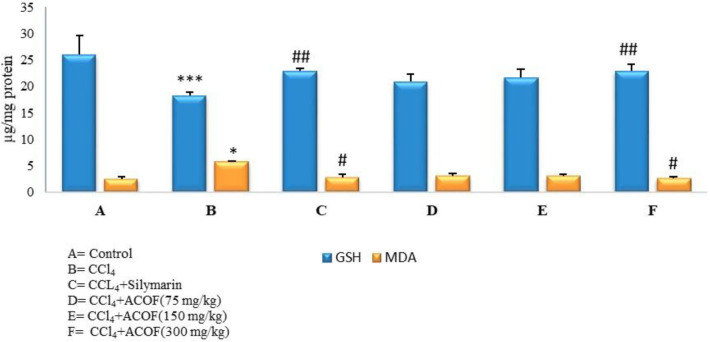
*In-vivo* antioxidant activity of the extract (µg/mL). ^*^P ≤ 0.05, ^***^P ≤ 0.001, *vs.* normal control.^ #^*P *≤ 0.05, ^##^*P *≤ 0.01, *vs.* CCl_4_ control

Hepatic GSH and MDA formation was completely restored to normal values by the treatment with the extract and Silymarin (Figure 1).

*Effect of ACOF on serum biochemical parameters*

The results of hepatoprotective effect of the extract at three doses (75, 150, and 300 mg/kg) on serum marker enzymes (AST, ALT, ALP, and LDH) and total bilirubin in CCl_4_-induced liver injury are shown in Table 1. CCl_4_-intoxicated rats showed significant rise in serum marker enzymes and total bilirubin; whereas the elevated levels of these parameters were significantly (*P *< 0.05) reduced in the groups treated with the extract also in the silymarin treated group when compared with CCl_4_-treated group. Treatment with different doses of the extract (75, 150, and 300 mg/kg) showed no significant difference with silymarin.

*Chromatographic separation*

**Figure 2:**
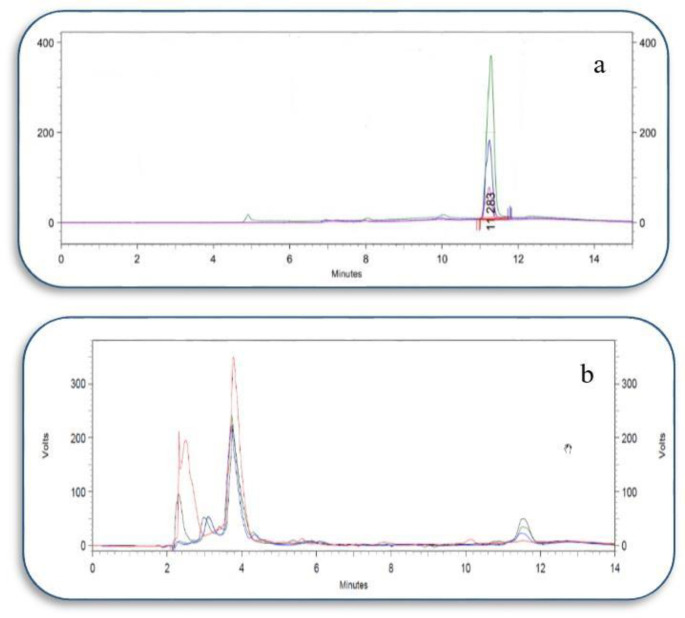
(a) chromatogram of three different concentrations of standard (ATP). (b) Chromatogram of blank (in red) and three different concentrations (olive:1, green: 0.5, and blue: 0.1 µg/mL) of ATP in isolated mitochondria. Peaks are overlaid on the same time axis

Through the chromatographic procedure explained in this study, ATP could be separated suitably from the other compounds existed in the isolated mitochondria (Figure 2).

Analytical data for ATP detection is summarized in Table 2.

*Method validation*

The method was validated for precision(inter/intra-day) and accuracy (recovery%) that are summarized in Table 3.

**Figure 3:**
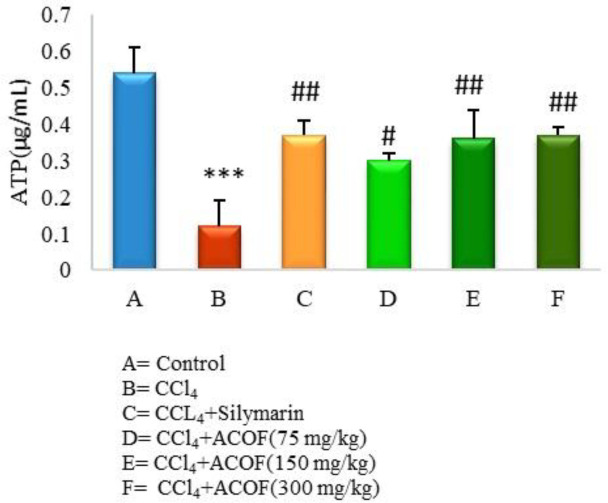
Effect of ACOF on hepatic mitochondrial ATP content for groups A-F against CCl_4_-induced liver damage. ^***^ *P *≤ 0.001, *vs.* normal control. ^#^*P *≤ 0.05, ^##^*P* ≤ 0.01, *vs.* CCl_4_ control

In order to consider the amount of endogenous ATP, a sample of the blank was injected and its concentration was calculated using a standard curve. Afterward, three different concentrations of ATP performed in triplicate were spiked to the blank sample and injected. The concentrations of them, determined from the standard curve, were compared with the used concentrations and the coefficient of variations (CV) was less than 10%. Moreover, the proposed method was used to determine the quantity of ATP in isolated mitochondria from different rat groups that are presented in Figure 3.

*Effect of ACOF on hepatic mitochondrial ATP content*

Results of the current study clearly revealed a significant decrease in the mitochondrial ATP in CCl_4_-intoxicated rats compared to control group (*P* < 0.05). Treatment with the ACOF in a dose dependent manner as well as silymarin significantly (*P* < 0.05) reversed this reduction while, the values of 150 and 300 mg/kg doses of the extract were comparable to the silymarin-treated group (Figure 3).

*Liver morphology and histopathology*

**Figure 4:**
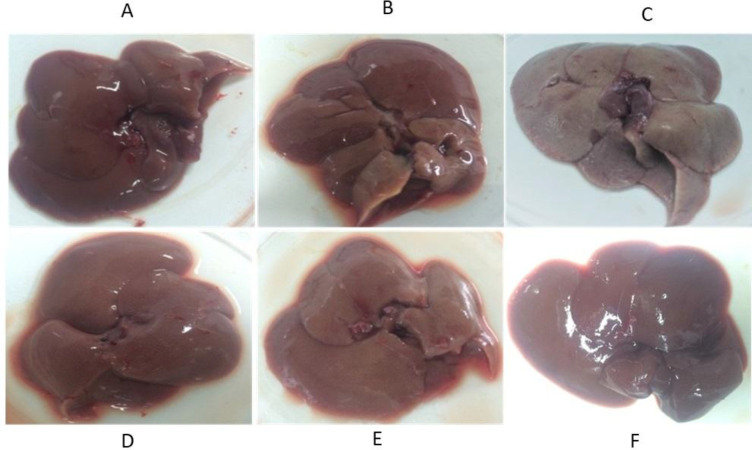
Morphological changes. (A) Liver section in the normal rat. (B) Silymarin treated rats, (C) CCl_4_-intoxicated rats, (D) Extract (75 mg/kg) treated rats, (D) Extract (150 mg/kg) treated rats, (D) Extract (300 mg/kg) treated rats

Livers of the CCl_4_ treated rats were larger and had more weight in comparison to the livers of the normal rats. In terms of color, in some places due to the dispersion of lymphocytic nuclei and also in some points due to the onset of necrosis in the tissue, there was a darkness in their tissues. The extract, in particular, at doses of 150 and 300 mg/kg, was able to minimize the apparent effects of toxicity (Figure 4).

**Figure 5:**
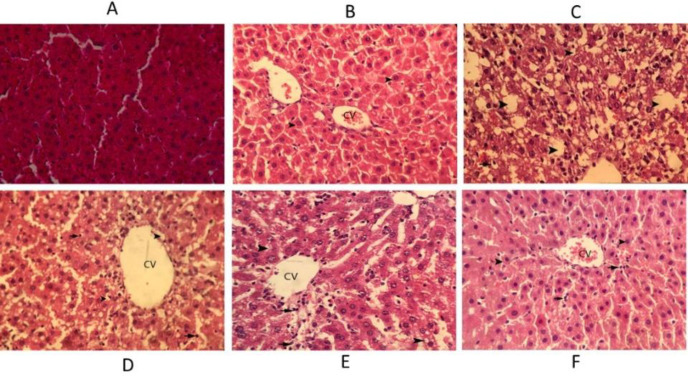
Hematoxylin–eosin staining of liver sections in rats. (A) Liver section in the normal rat. (B) Silymarin treated rats (C) CCl_4_-intoxicated rats, (D) Extract (75 mg/kg) treated rats, (E) Extract (150 mg/kg) treated rats, (F) Extract (300 mg/kg) treated rats, Arrows indicate hepatocyte degeneration, necrosis, and inflammatory cells infiltration, etc. Original magnification ×400. CV denotes central vein

In the control animals, the liver sections exhibited normal hepatic cells with well-preserved cytoplasm, prominent nucleus, and nucleolus, and also central vein. In the CCl_4_ treated rats severe fat degeneration in hepatocytes and necrosis of hepatocytes and infiltration of inflammatory cells are observed**.** In the rats treated with silymarin hepatocytes around the central veins was observed with a small amount of cellular swelling. In the rats treated with extract (75 mg/kg) around the central vein, fat degeneration occurred in hepatocytes and slight necrosis of hepatocytes and infiltration of inflammatory cells were observed. In the rats treated with extract (150 mg/kg) around the central vein, mild fatty degeneration in hepatocytes and infiltration of inflammatory cells occurred. In the rats treated with extract (300 mg/kg) hepatocytes around the central veins was observed with mild cellular inflammation and small infiltration of inflammatory cells (Figure 5).

The current study provides an additional strong evidence for hepatoprotective potential of ACOF. We induced hepatotoxicity in rats by CCl_4_ administration for 5 days to establish a situation in order to evaluate the effect of ACOF against mitochondrial ATP depletion in toxin-mediated liver injury. An attempt was performed to set up the HPLC method for determination of liver mitochondrial ATP levels. Consequently, an ion-pair reversed-phase HPLC method was used for this purpose. Mitochondrial dysfunction due to damage from CCl_4_–derived free radicals results in the loss of ATP supply which is considered as a causative factor in CCl_4_–induced cell death (27). In the current study, the reduction of ATP level in CCl_4_-treated rats by 78% demonstrated that mitochondria are an important target for CCl_4_ and bridged hepatic necrosis. Furthermore, ATP restoration by the treatment with ACOF demonstrated another powerful indicative for hepatoprotectivity of ACOF in addition to down regulating of liver enzymes serum levels. 

Liver damage by CCl_4_ is a complicated process that may turn into a defective circle while this organ is sustained by high doses or long term exposure of CCl_4_ which deprives the hepatocyte regenerative capacity. Hepatocellular regeneration process is also initiated; while there is sufficient energy supply within 24 h after subsiding of toxicant action (28). While in the current study the restoration of ATP level established the protection of hepatic mitochondria by ACOF, the initiation of liver recovery due to sustaining of mitochondrial ATP could be suggested. In the design of this study, we focused on the intake of ACOF that could be beneficial in mitochondrial liver injury. However, liver function test results beyond 24 h after the last CCl_4_ administration and also the measurement of liver regeneration parameters would be complementary to this study. 

Following CCl_4_ liver damage the serum level of enzymes like ALT, AST, ALP, and LDH by releasing them in the blood stream raises (29). In the current study CCl_4_ elevated all these enzymes significantly (*P *< 0.05) indicating severe hepatic cell necrosis. The elevated serum ALP also reflects the pathological alteration in biliary flow (30). In the current study the elevated level of serum ALP was in line with high level of serum bilirubin, an indicator of disease severity, in CCl_4 _treated rats. In this view, the reduction in serum levels liver enzymes as well as total bilirubin by ACOF revealed hepatoprotective activity of *quince *fruit against the toxic effect of CCl_4_, which was also supported by histological studies. 

CCl_4_ is biotransformed by hepatic microsomal cytochrome P450 to toxic metabolites, trichloromethyl (CCl_3_°) and trichloromethyl peroxy (CCl_3_OO°) radicals with high affinity for cell membrane lipids and different subcellular organelles especially mitochondria (31). It is well known that the hepatotoxic effect of CCl_4 _is due to the oxidative damage by the free radicals and antioxidant activity is a beneficial intervention in the protection against CCl_4_-induced liver damage (32-34). ACOF showed promising consequences against oxidative stress by reversion of increased lipid peroxidation and also maintaining hepatic glutathione. Its antioxidant capacity could be due to polyphenols,found in the extract (43.43 mg GAE/g). Although quince peel included a small portion of the fruit weight, polyphenolics contribution especially flavonoids of the peel, that was not removed in ACOF preparation, must be considered in the antioxidant capacity of ACOF. A previous study reported that quince fruit contains famous antioxidants such as caffeoylquinic acids, and rutin while quince peel besides caffeoylquinic acid contains other important flavonoidic antioxidants such as kaempferol 3- glucoside, quercetin 3-galactoside, and kaempferol-3-rutinoside (35). However, in the current study due to lack of data related to the effect of the individual antioxidant compounds isolated from ACOF we could not determine constitutes responsible for hepatoprotective activity of the extract. Further studies to identify active principle (s) responsible for hepatoprotective activity and to find out synergy among different compounds present in quince fruit would be complementary to this study. 

**Table 1:** Effect of ACOF on serum biochemical parameters against CCl_4_ induced liver damage

	**A**	**B**	**C**	**D**	**E**	**F**
AST (IU/L)	818.5 ± 165.7	1614.8 ± 286.8^***^	1014.3 ± 234.8^##^	960 ± 128.8^##^	943.4 ± 81.2^##^	1070.5 ± 76^##^
ALT (IU/L)	64 ± 7.2	2210 ± 97.1^***^	124.5 ± 26.4^###^	126.3 ± 57.8^###^	146.8 ± 46^###^	161.2 ± 55.3^###^
ALP (IU/L)	274.4 ± 47	1917 ± 208.8^***^	132.5 ± 61.1^###^	185 ± 50.6^###^	183.8 ± 51.4^###^	207.5 ± 23.4^###^
LDH (IU/L)	1040.3 ± 228	2241 ± 221^*^	489.66 ± 120^##^	868.7 ± 233^##^	754.5 ± 267^#^	876 ± 277^##^
Total Bilirubin (mg/dL)	0.14 ± 0.02	0.45 ± 0.07^**^	0.2 ± 0.04^##^	0.23 ± 0.04	0.16 ± 0.05^##^	0.17 ± 0.03^##^

A: Control; B: CCl_4_; C: CCl_4_+ silymarin; D: CCl_4_+ ACOF (75 mg/kg); E: CCl_4_+ ACOF (150 mg/kg); F: CCl_4_+ ACOF (300 mg/kg).

Values are presented as mean ± SD, ^*^P ≤ 0.05, ^**^ *P*≤0.01, ^***^ *P *≤ 0.001, *vs*. normal control. ^#^*P *≤ 0.05, ^##^*P *≤ 0.01,^ ###^*P *≤ 0.01 *vs.* CCl_4_ control.

**Table 2:** Analytical data for determination of ATP

**Analyte**	**Retention time** ** (min)**	**slope**	**intercept**	**Range (µg/mL)**	**Linearity** ** (r** ^2^ **)**	**LOD (µg/mL)**	**LLOQ (µg/mL)**
ATP	11.3	230198	4031	0.1-1	0.9994	0.04	0.1

**Table 3:** validation parameters of ATP analysis in isolated mitochondria

**Concentration added** **(µg/mL)**	**Concentration measured (mean ± SD)**		**Recovery (%)**
	**Intra-day (CV%)**	**Inter-day (CV%)**	
1	1.08 ± 0.04 (3.7)	1.12 ± 0.03 (2.6)	107.5
0.5	0.48 ± 0.02 (4.2)	0.53 ± 0.03 (5.6)	96.7
0.1	0.11 ± 0.008 (7.2)	0.13 ± 0.01 (7.6)	111.2

## Conclusion

Quantifying liver mitochondrial ATP content along with the other , including biochemical and oxidative stress related factors in response to CCl_4 _yielded greater insight into the mechanism of hepatoprotectivity of quince fruit. Furthermore, from the data presented here it could be concluded that the protective effect of ACOF on mitochondrial dysfunction could have an important role in its hepatoprotective activity against CCl_4_-induced hepatotoxicity. 

## References

[1] Wills PJ, Asha VV (2006). Protective effect of Lygodium flexuosum (L) Sw extract against carbon tetrachloride-induced acute liver injury in rats. J. Ethnopharmacol..

[2] Chandan BK, Saxena AK, Shukla S, Sharma N, Gupta DK, Suri KA, Suri J, Bhadauria M, Singh B (2007). Hepatoprotective potential of Aloe barbadensis Mill against carbon tetrachloride induced hepatotoxicity. J. Ethnopharmacol..

[3] Singh B, Saxena AK, Chandan BK, Bhardwaj V, Gupta VN, Suri OP, Handa SS (2001). Hepatoprotective activity of indigtone--a bioactive fraction from Indigofera tinctoria Linn. Phytother. Res..

[4] Joshi PP, Patil SD, Silawat N, Deshmukh PT (2011). Effect of Tridax procumbens (Linn ) on bile duct ligation-induced liver fibrosis in rats. Nat. Prod. Res..

[5] Jain S, Dixit VK, Malviya N, Ambawatia V (2009). Antioxidant and hepatoprotective activity of ethanolic and aqueous extracts of Amorphophallus campanulatus Roxb. tubers. Acta Pol. Pharm..

[6] Fattouch S, Caboni P, Coroneo V, Tuberoso CI, Angioni A, Dessi S, Marzouki N, Cabras P (2007). Antimicrobial activity of Tunisian quince (Cydonia oblonga Miller) pulp and peel polyphenolic extracts. J. Agric. Food Chem..

[7] Pacifico S, Gallicchio M, Fiorentino A, Fischer A, Meyer U, Stintzing FC (2012). Antioxidant properties and cytotoxic effects on human cancer cell lines of aqueous fermented and lipophilic quince (Cydonia oblonga Mill ) preparations. Food Chem. Toxicol..

[8] Zhou W, Abdusalam E, Abliz P, Reyim N, Tian S, Aji Q, Issak M, Iskandar G, Moore N, Umar A (2014). Effect of Cydonia oblonga Mill ruit and leaf extracts on blood pressure and blood rheology in renal hypertensive rats. J. Ethnopharmacol..

[9] Zhou W, Abdurahman A, Umar A, Iskander G, Abdusalam E, Berké B, Bégaud B, Moore N (2014). Effects of Cydonia oblonga Miller extracts on blood hemostasis, coagulation and fibrinolysis in mice, and experimental thrombosis in rats. J. Ethnopharmacol..

[10] Minaiyan M, Ghannadi A, Etemad M, Mahzouni P (2012). A study of the effects of Cydonia oblonga Miller (Quince) on TNBS-induced ulcerative colitis in rats. Res. Pharm. Sci..

[11] Huber R, Stintzing FC, Briemle D, Beckmann C, Meyer U, Gründemann C (2012). In-vitro antiallergic effects of aqueous fermented preparations from Citrus and Cydonia fruits. Planta Med..

[12] Ghafourian M, Tamri P, Hemmati A (2015). Enhancement of human skin fibroblasts proliferation as a result of treating with quince seed mucilage. Jundishapur J. Nat. Pharm. Prod..

[13] Aslan M, Orhan N, Orhan DD, Ergun F (2010). Hypoglycemic activity and antioxidant potential of some medicinal plants traditionally used in Turkey for diabetes. J Ethnopharmacol..

[14] Gholami S, Hosseini MJ, Jafari L, Omidvar F, Kamalinejad M, Mashayekhi V, Hosseini SH, Kardan A, Pourahmad J, Eskandari MR (2017). Mitochondria as a Target for the Cardioprotective Effects of Cydonia oblonga Mil and Ficus carica L in Doxorubicin-Induced Cardiotoxicity. Drug Res Stuttg).

[15] Khademi F, Danesh B, Mohammad Nejad D, Soleimani Rad J (2013). The comparative effects of atorvastatin and quince leaf extract on atherosclerosis. Iran. Red Crescent Med. J..

[16] Mirmohammadlu M, Hosseini SH, Kamalinejad M, Esmaeili Gavgani M, Noubarani M, Eskandari MR (2015). Hypolipidemic, hepatoprotective and renoprotective effects of Cydonia oblonga mil fruit in streptozotocin-induced diabetic Rats. Iran. J. Pharm. Res..

[17] Adiban H, Shirazi FH, Gholami S, Kamalinejad M, Hosseini SH, Noubarani M, Eskandari MR (2019). Chemopreventive effect of quince (Cydonia oblonga Mill ) fruit extract on hepatocellular carcinoma induced by diethylnitrosamine in rats. Int. Pharm. Acta.

[18] Aliasl F, Toliyat T, Mohammadi A, Minaee B, Samadi N, Aliasl J, Sadeghpour O (2016). Medicinal properties of Cydonia oblonga mill fruit (pulp and peel) in Iranian traditional medicine and modern phytothrapy. Trad. Integr. Med..

[19] Hernández-Muñoz R, Díaz-Muñoz M, Suárez-Cuenca JA, Trejo-Solís C, López V, Sánchez-Sevilla L, Yáñez L, De Sánchez VC (2001). Adenosine reverses a preestablished CCl4 induced micronodular cirrhosis through enhancing collagenolytic activity and stimulating hepatocyte cell proliferation in rats. Hepatology.

[20] Blainski A, Lopes GC, Pallazo de Mello JC (2013). Application and analysis of the folin ciocalteu method for the determination of the total phenolic content from Limonium brasiliense L. Molecules.

[21] Chang C, Yang M, Wen H, Chern J (2002). Estimation of total flavonoid content in propolis by two complementary colorimetric methods. J. Food Drug Anal..

[22] Wang, M, Li J, Rangarajan M, Shao Y, Lavoie E, Huang T, Ho CT (1998). Antioxidative phenolic compounds from sage (Salvia officinalis). J. Agric. Food Chem..

[23] Noubarani M, Khayat SA, Mafinezhad R, Mohebbi S, Mohammad K, Andalib S, Kardan A, Eskandari MR (2016). Protective effect of Cydonia oblonga Mill fruit on carbon tetrachloride-induced hepatotoxicity. Planta Med..

[24] Lambowitz AM (1979). Preparation and analysis of mitochondrial ribosomes. Methods Enzymol..

[25] Smith MT, Thor H, Hartizell P, Orrenius S (1982). The measurement of lipid peroxidation in isolated hepatocytes. Biochem. Pharmacol..

[26] Riener C, Kada G, Gruber HJ (2002). Quick measurement of protein sulfhydryls with Ellman’s reagent and with 4,4-dithiodipyridine. Anal. Bioanal. Chem..

[27] Nieminen AL, Qian T, Trost LC, Elmore SP, Nishimura Y, Crowe RA, Cascio WE, Bradham CA, Brenner DA, Herman B (1998). The mitochondrial permeability transition in cell death: a common mechanism in necrosis, apoptosis and autophagy. Biochim. Biophys. Acta.

[28] Mehendale HM (1991). Role of hepatocellular regeneration and hepatolobular healing in the final outcome of liver injury — a two-stage model of toxicity. Biochem. Pharmacol..

[29] Asha VV (2001). Preliminary studies on the hepatoprotective activity of Mamordica subangulata and Naragamia alata. Indian J. Pharmacol..

[30] Ploa, GL, Hewitt WR, Wallace Hayes A (1989). Detection and evaluation of chemically induced liver injury. Principles and Methods of Toxicology.

[31] Boll M, Weber LW, Becker E, Stampfl A (2001). Mechanism of carbon tetrachloride-induced hepatotoxicity Hepatocellular damage by reactive carbon tetrachloride metabolites. Z. Naturforsch. C.J.Biosci..

[32] Shih PH, Hsu CL, Yen GC (2007). Hepatoprotection of tea seed oil (Camellia oleifera Abel ) against CCl4-induced oxidative damage in rats. Food Chem. Toxicol..

[33] Srivastava A, Shivanandappa T (2006). Hepatoprotective effect of the aqueous extract of the roots of Decalepis hamiltonii against ethanol-induced oxidative stress in rats. Hepatol. Res..

[34] Jain A, Soni M, Deb L, Jain A, Rout SP, Gupta VB, Krishna KL (2008). Antioxidant and hepatoprotective activity of ethanolic and aqueous extracts of Momordica dioica Roxb. leaves. J. Ethnopharmacol..

[35] Silva BM, Andrade PB, Ferreres F, Domingues AL, Seabra RM, Ferreira MA (2002). Phenolic profile of quince fruit (Cydonia oblonga Mil ) (pulp and peel). J. Agric. Food Chem..

